# Non-Destructive Detection Pilot Study of Vegetable Organic Residues Using VNIR Hyperspectral Imaging and Deep Learning Techniques

**DOI:** 10.3390/s21092899

**Published:** 2021-04-21

**Authors:** Youngwook Seo, Giyoung Kim, Jongguk Lim, Ahyeong Lee, Balgeum Kim, Jaekyung Jang, Changyeun Mo, Moon S. Kim

**Affiliations:** 1 Department of Agricultural Engineering, National Institute of Agricultural Sciences, 310 Nongsaengmyeong-ro, Deokjin-gu, Jeonju 54875, Korea; giyoung@korea.kr (G.K.); limjg@korea.kr (J.L.); lay117@korea.kr (A.L.); bgkim@korea.kr (B.K.); jkjang1052@korea.kr (J.J.); 2 Department of Biosystems Engineering, Kangwon National University, 1 Kangwondaehak-gil, Chuncheon 24341, Gangwon-do, Korea; cymoh100@kangwon.ac.kr; 3 Interdisciplinary Program in Smart Agriculture, Kangwon National University, Chuncheon 24341, Gangwon-do, Korea; 4 Environmental Microbial and Food Safety Laboratory, Beltsville Agricultural Research Center, Agricultural Research Service, USDA, 10300 Baltimore Avenue, Beltsville, MD 20705, USA

**Keywords:** VNIR hyperspectral imaging, vegetable organic residue, stainless-steel surface, detection, classification, food contaminants

## Abstract

Contamination is a critical issue that affects food consumption adversely. Therefore, efficient detection and classification of food contaminants are essential to ensure food safety. This study applied a visible and near-infrared (VNIR) hyperspectral imaging technique to detect and classify organic residues on the metallic surfaces of food processing machinery. The experimental analysis was performed by diluting both potato and spinach juices to six different concentration levels using distilled water. The 3D hypercube data were acquired in the range of 400–1000 nm using a line-scan VNIR hyperspectral imaging system. Each diluted residue in the spectral domain was detected and classified using six classification methods, including a 1D convolutional neural network (CNN-1D) and five pre-processing methods. Among them, CNN-1D exhibited the highest classification accuracy, with a 0.99 and 0.98 calibration result and a 0.94 validation result for both spinach and potato residues. Therefore, in comparison with the validation accuracy of the support vector machine classifier (0.9 and 0.92 for spinach and potato, respectively), the CNN-1D technique demonstrated improved performance. Hence, the VNIR hyperspectral imaging technique with deep learning can potentially afford rapid and non-destructive detection and classification of organic residues in food facilities.

## 1. Introduction

Contamination inspection of food facilities is indispensable to ensure food safety. While raw agricultural products are consumed without any processing, certain procedures, such as peeling, shredding, cutting, trimming, extruding, and sanitizing, require processed agricultural products [1]. After the completion of processing operations, certain amounts of organic materials may remain on the blades, cracks, or crevices in the facilities. This can produce infectious foodborne bacteria. Therefore, several studies investigated the risk of infection from food processing machines. Researchers collected bacterial samples from fresh-cut processing facilities after sanitization. The collected samples were incubated on a general growth media for 24 h, from which the mesophilic and psychrotrophic bacteria were isolated and identified. The studies reported that approximately 30% of more than 1000 isolated pathogen samples can potentially provide environmental protection to the foodborne pathogens [2]. Therefore, contamination of fresh-cut products can occur at any time during the processing of agricultural products [3,4].

Conventionally, the hygiene assessment in food processing facilities is performed by plating and incubating the samples on growth media for 24–48 h [5]. Typically, the cultivation of microorganisms is time-consuming and requires trained operators to perform intensive lab work. However, several rapid and non-destructive organic molecular component detection and classification techniques exist to evaluate food safety, particularly to ensure hygiene and sanitation in mass food processing facilities.

For decades, X-ray, near-infrared spectroscopy, and computer vision have contributed to the development of non-destructive safety inspection technologies [6,7]. For instance, Fourier transform near-infrared (FT-NIR) and FT-IR spectroscopic methods identified the unexpected contamination of onion powder by starch [8], and Raman spectroscopic technology differentiated between fake and real eggs [9]. Hyperspectral imaging (HSI) is an emerging technology that uses a data cube with spectral and spatial data to analyze organic residues. For instance, the melamine content in infant formulas was detected and isolated using HSI technology [10]. Furthermore, multiple HSI modalities, including visible and near-infrared (VNIR, 400–1000 nm) and short-wavelength IR waves (900–1700 nm), were used to detect and classify mislabeled fish fillets [11]. Additionally, multispectral laser-induced fluorescence imaging detected different dilutions of animal fecal matter on apples, and three dilutions of 1:2, 1:20, and 1:200 detected approximately 80% of fecal matter within 24 h after the application of the technique. However, the detection accuracy lowered when the apples were brushed and washed; the fecal matter detected were 100%, 30%, and 0% for the 1:2, 1:20, and 1:200 dilutions, respectively [12].

Conversely, the deep learning method performs reliably with multidimensional data, including hyperspectral data [13,14]. Convolutional neural networks (CNNs) exhibit accurate classification and feature extraction with 1D, 2D, and 3D data [15,16,17,18]. In particular, CNN-1D is considered ideal for processing signals and prediction models, such as medical electrocardiography signals [19], environmental sounds [20], and human activity recognition [21]. Additionally, CNN-1D classified the spectral data of foodborne bacteria using hyperspectral microscope imaging technology with higher accuracy (90%) than that of the machine learning methods [22].

This study aimed to develop a non-destructive technique to detect and classify the concentration of biological residues in spinach and potato juices on stainless-steel surfaces. The proposed technique acquired the 3D hypercube data using a VNIR HSI system. Furthermore, a CNN-1D and several chemometric methods demonstrated the detection and classification results of the potato and spinach droplets diluted to six different concentrations.

## 2. Materials and Methods

### 2.1. Sample Preparation

We purchased fresh potato (*Solanum tuberosum*) and spinach (*Spinacia oleracea*) from a local market to prepare the diluted residues. Initially, the products were cut and squeezed to extract the juice. The diluted juice samples were then placed on stainless-steel plates and dried for 24 h. The experimental analysis was performed on six samples, namely, a 100% undiluted original fresh juice and five dilutions of potato and spinach, at 20% (1:5), 10% (1:10), 5% (1:20), 2% (1:50), and 1% (1:100), prepared by adding distilled water to the juice. Approximately 10 μL of the diluted solutions were placed on the plate with 15 rows and 2 replicates using a pipette. Thus, the total number of diluted droplets was 90 (6 dilutions × 15 repeats) on the stainless-steel plate.

### 2.2. VNIR HSI System and Data Acquisition

The HSI system comprises a 14-bit electron multiplying charge-coupled device (EMCCD) camera (Luca R DL-604M, Andor Technology, South Windsor, CT, USA) with a shutter speed of 80 ms, coupled with a C-mount objective lens (F1.9 35 mm compact lens, Schneider Optics, Van Nuys, CA, USA). The VNIR spectra in the range of 400–1000 nm were acquired using a spectrophotometer (VNIR Hyperspec, Headwall Photonics, Inc.; Fitchburg, MA, USA) that was combined with the EMCCD camera. Additionally, two halogen lamps provided the lighting system. Each sample plate was placed on a linear motorized platform (Velmex, Inc.; Bloomfield, NY, USA) to convey the samples using a lab-built HSI system (ARS, USDA, Beltsville, MD, USA). The sample plates placed on the moving platform acquired the VNIR 3D hypercube data of the diluted droplets using a line-scan camera. Initially, the dark and white references were captured and applied to calibrate the raw images before acquiring the hyperspectral images. The VNIR HSI data contained images of 1000 × 1004 pixels in size and 128 bands in the range of 400–1000 nm.

### 2.3. Region of Interest (ROI) Selection

**Figure 1:**
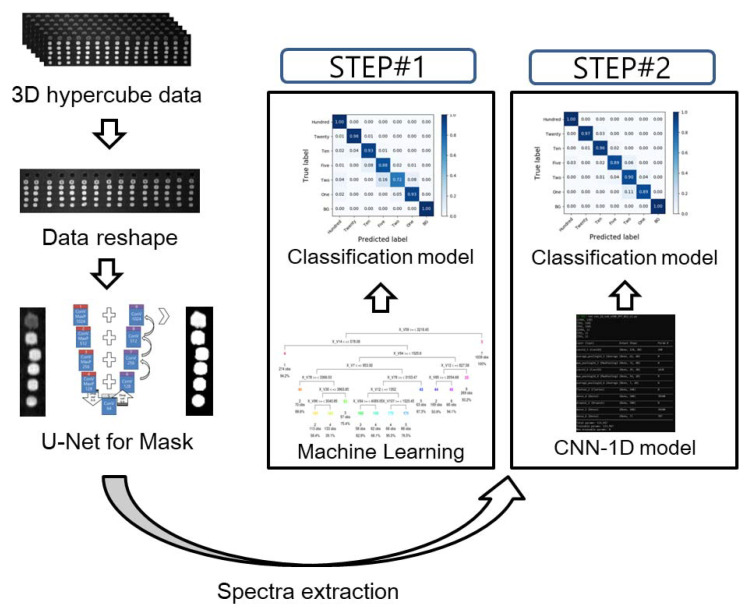
Flow chart of the data processing and schematic architecture of a 1D convolutional neural network. In order to develop the classification model, STEP#1 is using chemometric methods and STEP#2 is using the CNN-1D algorithm.

Figure 1 illustrates the flow chart of the image processing and the development of the classification models. To extract spectral data from the raw VNIR HSI images, an optimal region of interest (ROI) must be selected. The Otsu algorithm, principal component analysis (PCA), and U-net demonstrated the ROI selection results.

The Otsu method is a well-known image binarization algorithm that uses an image thresholding technique [23]. In this study, the threshold values for spinach and potato were determined as 110 and 98, respectively. On the other hand, PCA calculates the correlation with input data. However, the spectral data were extracted based on the ROI selected using the U-net method. Additionally, stainless-steel background (BG) spectral data were randomly selected from 12 regions apart from the sample droplets. To enhance the quality of the selected band image, a median filter and an image sharpening technique were applied. The dilutions of the potato and spinach droplets were considered as “Hundred”, “Twenty”, ”Ten”, ”Five”, ”Two”, and ”One” for the classification, corresponding to the dilutions of 100% (original fresh juice), 20% (1:5), 10% (1:10), 5% (1:20), 2% (1:50), and 1% (1:100), respectively.

### 2.4. U-Net for Feature Segmentation

**Figure 2:**
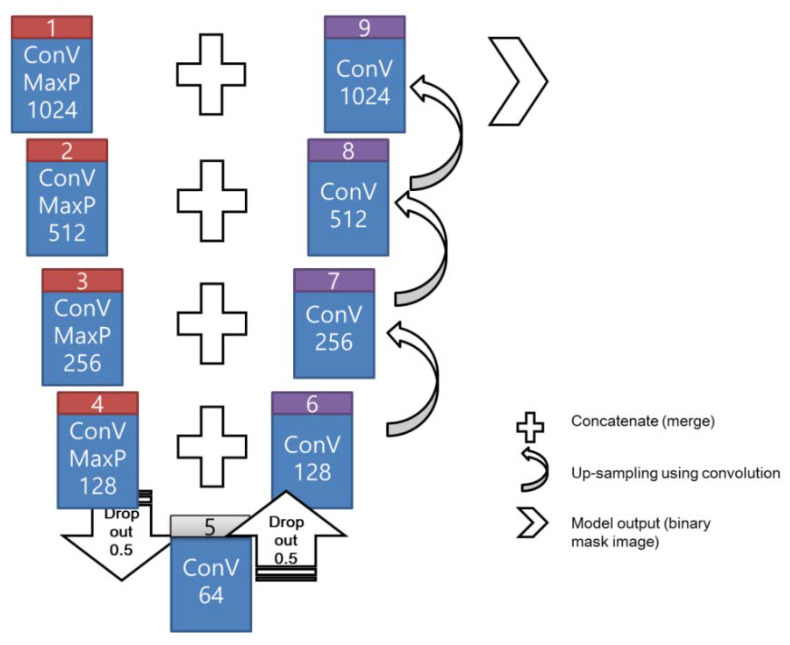
Schematic flowchart of the U-net architecture for image masking.

Figure 2 illustrates the schematic of the U-net architecture applied in the image processing. U-net is a CNN developed for biomedical image segmentation [24], wherein the architecture comprises the encoding and decoding procedures. The left (box# 1–4) and right (box# 6–9) portions in the figure represent the encoding and decoding procedures, respectively. A convolution kernel exists throughout the procedure. The data extraction and compression occurs during encoding. Additionally, this model adopted the rectified linear unit (ReLU) as the activation function and a 2 × 2 max-pooling kernel with a dropout coefficient of 0.5 to achieve dimensionality reduction in the data. The input data were downsized by a quart during the encoding procedure and subsequently merged during up-sampling in the initial convolution of the decoding procedure. The concatenate function merges the dropout and up-sampling convolution.

In the decoding process, a 2 × 2 convolution layer was implemented to reconstruct a new feature map rather than the max-pooling layer. Moreover, the concatenate function (merging operation) was implemented with the corresponding feature maps (results of the dropout or convolution) from the encoding process to develop a feature map in each decoding layer. For instance, two 128-channel feature maps (from boxes #4 and #6) were merged using the concatenate function to generate a 256-channel feature map (box #7). At the final layer (box #9), a 1 × 1 convolution kernel transformed the final feature map to yield the output (mask image in binary mode).

### 2.5. Development of the Classification Model

The classification model was developed based on two strategies (Figure 1). As indicated in the figure, STEP #1 constitutes the chemometric methods that involve multivariate analysis methods and machine learning algorithms. Conversely, STEP #2 uses the CNN-1D algorithm. Table 1 presents the detailed model architecture and specifications.

Both linear and non-linear multivariate classification methods, such as linear discriminant analysis (LDA), partial least squares discriminant analysis (PLS-DA), support vector machine (SVM), decision tree (DT), least squares support vector machine (LSSVM), and random forest (RF), were used to analyze the results [25,26]. RF is an ensemble algorithm of DTs {$T_{1}\left(X\right),\;\ldots ,\;T_{n}\left(X\right)$}, wherein X = {$x_{1},\;\ldots ,\;x_{n}$} is an *n*-dimensional vector of properties associated with a dependent variable (spectrum of diluted droplets). The tree ensemble yields N outputs {$Y_{1}=T_{1}\left(X\right),\;\ldots ,\;Y_{n}=T_{n}\left(X\right)$}, wherein $Y_{n},\;n=$1, …, N, represent the class predicted by the *n*-th tree for the input data [27,28]. The classification results were obtained using six preprocessing methods. No-P denotes no pre-processing; D1 and D2 are the 1st and 2nd derivatives, respectively, based on the Savitzky–Golay algorithm; MSC, MA, and NM represent the multiplicative scatter correction, moving average, and normalization, respectively. The accuracy and Cohen’s kappa coefficient were used to show the results of the used classificaiton methods based on the confusion matrix [29]. Cross-validation was performed to evaluate the accuracy of the classification models using the leave-one-out (LOO) method. All classification algorithms and pre-processing methods were coded using R (Ver. 3.6.2.), the statistical open-source environment and language. The model was developed using multiple classification packages, such as caret (Ver. 6.0–85), e1071 (Ver. 1.7–3), rpart (Ver. 4.1–15), kernlab (Ver. 2004), and randomForest (Ver. 4.6). The Otsu algorithm was performed using ImageJ (Ver. 1.53c), which is an open-source scientific image processing program.

To classify the spectral data obtained from the diluted residues, a CNN-1D model was developed based on the architecture and parameters presented in Table 1. The optimized CNN-1D algorithm comprises convolution, average pooling, max-pooling, dropout, and output. The activation function uses ReLU to produce an image from a linear model. Both average pooling and max-pooling are applied to reduce the dimensionality of the spectral data. The total number of parameters and repeated epochs were 123,967 and 5000, respectively. However, these values can vary depending on the state of convergence. Deep learning classification was performed using Python (Ver. 3.7.4), and the corresponding packages included Tensorflow (Ver. 2.2.0), Keras (Ver. 2.3.1), and Scikit-image (Ver. 0.17.1). The CNN-1D frameworks were developed and trained on a computer equipped with an i7-8750H (CPU), GeForce GTX1050 Ti (GPU), and 16 GB memory.

## 3. Results and Discussion

### 3.1. ROI Segmentation

**Figure 3:**
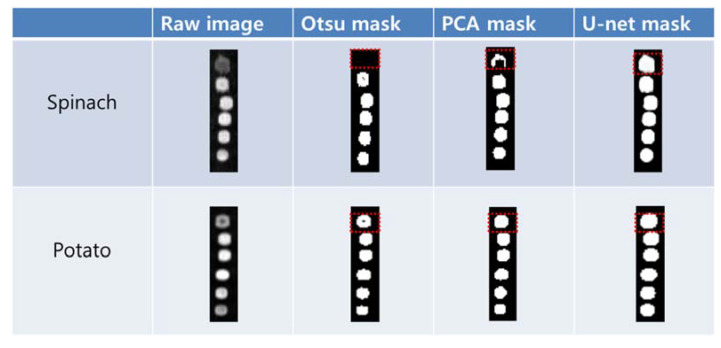
Target segmentation results of the spinach and potato residue droplets. The mask generated by U-net had less loss of the target image pixels.

We used a mask image to select the ROIs of the potato and spinach residues automatically from the corrected sample images. The mask image was developed to observe the segmentation results in the column regions (20 × 86 pixels) of six dilutions obtained from the 15 repeated raw images (391 × 86 pixels). Figure 3 depicts the mask images of the spinach and potato residues obtained from the Otsu algorithm, PCA, and U-net methods along with the sample raw images. In the case of spinach, the color of the residue Hundred (100%) in the raw image is different from that of the other diluted residues. While the Otsu and PCA masks segmented the entire sample in the five diluted residues, U-net produced all the samples with limited loss in image pixels. Although the droplets of potato exhibited different intensities between the residues of Hundred (100%) and One (1%) in the raw image, PCA and U-net produced appropriate results.

### 3.2. VNIR Spectral Characteristics

**Figure 4:**
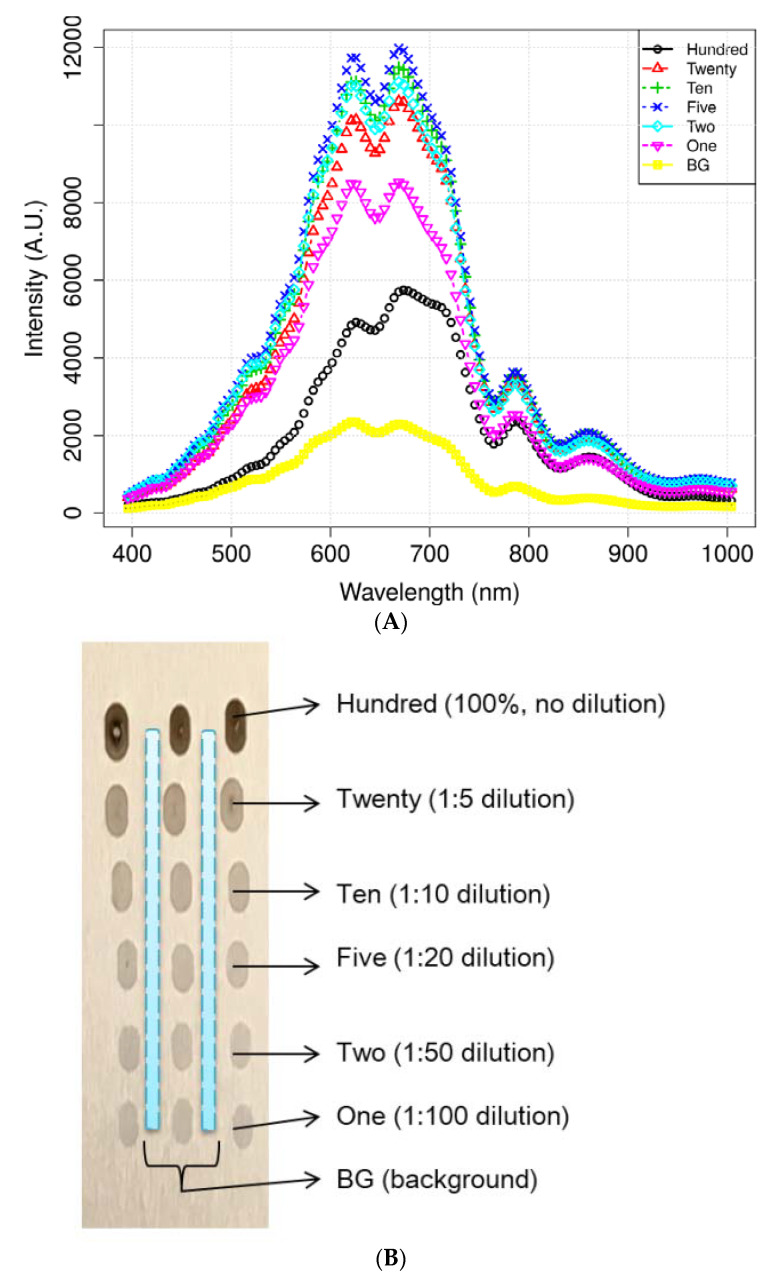

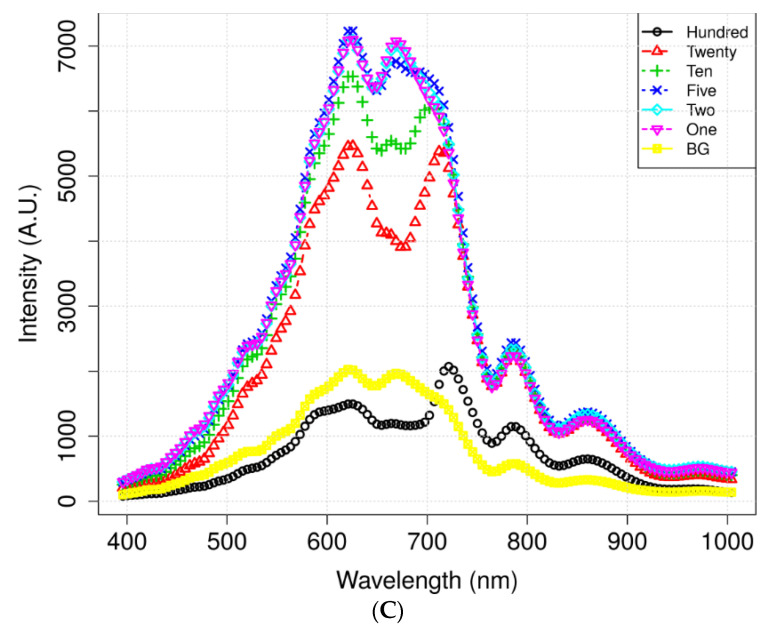
Mean spectrum data of (**A**) potato and its locational (**B**) residues according to the dilutions and background (BG) pixels. (**C**) Mean spectra of spinach.

**Figure 5:**
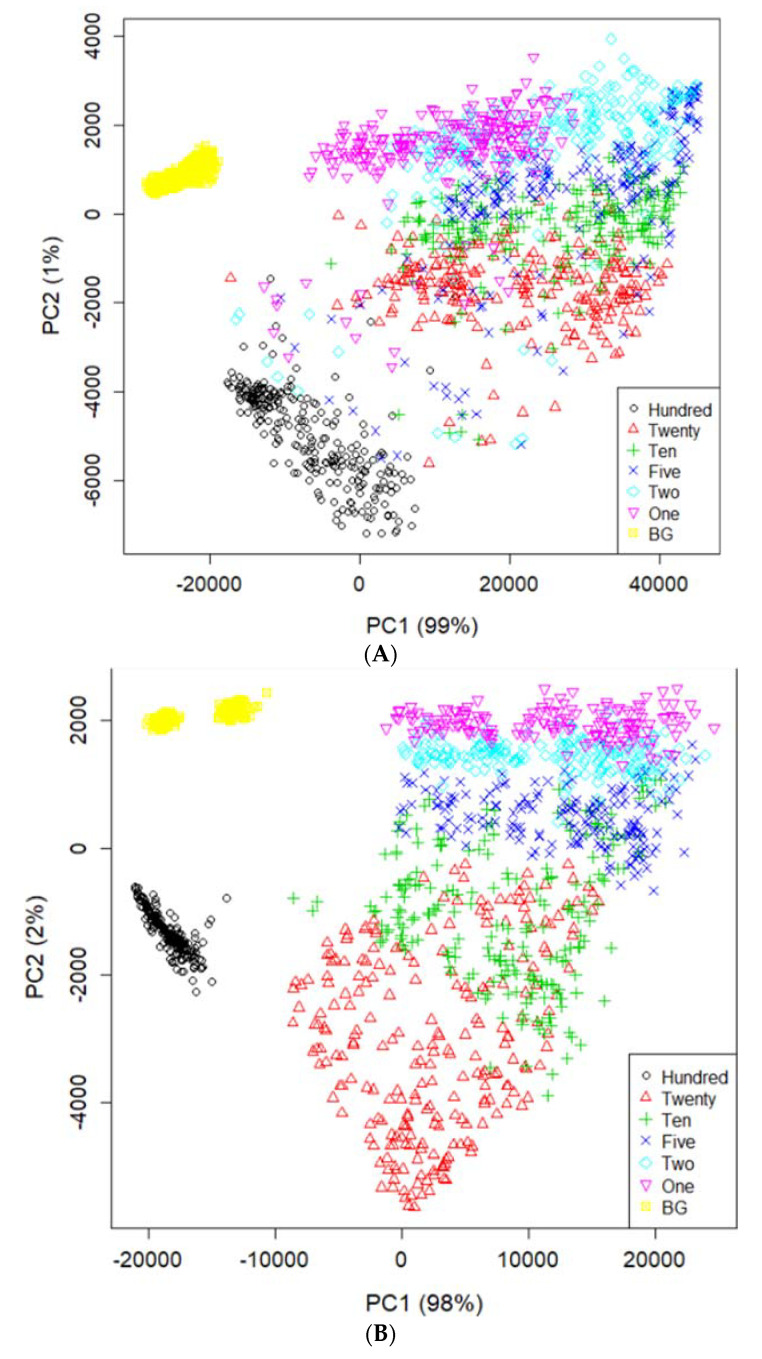
PCA image on PC1 and PC2 space using the spectral data of potato (**A**) and spinach (**B**) from the residues and background ROI.

Figure 4 illustrates the mean spectra extracted from the HSI data obtained from the residues of the potato juice and the stainless-steel BG. The colored image depicts six dilutions of potato residues and the extracted region of the BG spectrum. The peaks in the mean spectrum were observed at 625, 720, 785, and 860 nm. Typically, most of the selected bands within the VNIR regions are associated with physiological substances, such as the CH, NH, and OH stretching, in the vibrational spectrum. For instance, absorption of anthocyanin and carotenoid occur at 650 and 680 nm, respectively [30,31]. Spectral bands at 690–710 nm and 760–800 nm represent the total chlorophyll bands, whereas the absorption bands at 705, 842, and 920 nm are associated with carbohydrates [31]. The band at 995 nm represents the 2nd vibration of the NH bonds in proteins or amino acids, whereas that at 880 nm constitutes the 3rd overtone absorption of CH. Additionally, the band relates to the 2nd overtone absorption of the OH and NH bonds at 750–900 nm and 962–1000 nm, respectively [32]. Figure 5 illustrates the score scattering attributes demonstrated in the principal components (PCs) during intuitive data analysis. We assigned seven colors to the residues based on the dilutions of potato and the BG surface. The first PC (PC1) denotes the variance of the potato residues at six dilutions and the BG spectral data as 99% and 1%, respectively. Conversely, the second PC (PC2) indicates the variance of the spinach residues at six dilutions and the BG spectral data as 98% and 2%, respectively. The original juice (Hundred, no dilution) was easily distinguishable in the PC score plots (black, circle). Moreover, a class of BG (yellow, square) was isolated from the diluted residues. Figure 5 shows that the low-dilution residues (<10%) of potato demonstrated overlapping clusters.

### 3.3. Classification Results

Six multivariate analysis methods and machine learning algorithms were used to classify the diluted residues on the stainless-steel surface. Table 2 and Table 3 present the classification results of the potato and spinach residues, respectively, considering the accuracy (*A*) and kappa coefficient (*K*) in the classification models.

**Figure 6:**
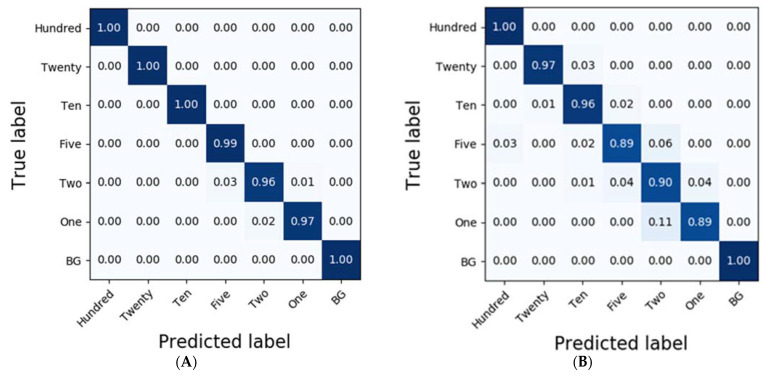
Confusion matrix of the classification result for potato according to the dilution of the residues using CNN-1D. The calibration result (*Ac* = 0.99) is (**A**) and the validation result (*Av* = 0.94) is (**B**).

In the case of potato residues, LSSVM and RF exhibited higher accuracies than 0.86 based on the pre-processing methods, such as No-P, D1, MA, and NM. Additionally, LDA demonstrated reasonable classification results at an accuracy of 0.83; however, the accuracies of PLS-DA and DT were less than 0.77. Conversely, the classification results obtained from SVM were of the highest accuracy at 0.90 (Table 2), and the detailed results for each of the diluted residues were 1.0, 0.89, 0.87, 0.93, 0.71, 0.94, and 0.95 for Hundred, Twenty, Ten, Five, Two, One, and BG, respectively. However, CNN-1D demonstrated improved accuracies compared to those of SVM in each of the residues at 1.0, 0.97, 0.96, 0.89, 0.90, 0.89, and 1.0 (Figure 6).

**Figure 7:**
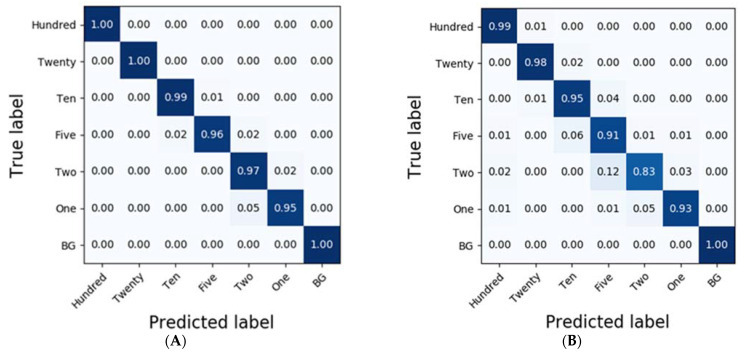
Confusion matrix of the classification result for spinach dilutions of the residues using CNN-1D. The calibration result (*Ac* = 0.98) is (**A**) and the validation result (*Av* = 0.94) is (**B**).

In the case of spinach residues, SVM exhibited the most accurate classification results with an accuracy of 0.92 (Table 3). Moreover, the accuracies of the classification results obtained from RF were higher than 0.9 in the case of D1 and MSC. While SVM classified the results of each of the residues at accuracies of 1.0, 0.96, 0.90, 0.88, 0.81, 0.85, and 0.98, CNN-1D demonstrated improved accuracies of 0.99, 0.98, 0.95, 0.91, 0.83, 0.93, and 1.0 (Figure 7).

To compare the results of the classification models, spectral data analysis was performed using the CNN-1D algorithm. Six diluted residues were classified, and the results were presented using a confusion matrix. In this study, the numbers of training epochs and parameters were 500 and 123,967, respectively. The mean absolute error and the loss were 0.0262 and 0.0093, respectively, after the model was trained. Figure 6 and Figure 7 depict the confusion matrices representing the prediction accuracies of the developed CNN-1D model applied to the validation dataset. While Figure 6 depicts the classification and validation results of the potato residues (*Ac* = 0.99, *Av* = 0.94), Figure 7 presents those of the spinach residues (*Ac* = 0.98, *Av* = 0.94). These results indicate that the CNN-1D improves the classification accuracy by 2–4% from 0.92 and 0.90 in the case of potato and spinach, respectively, using the chemometric method

## 4. Conclusions

To detect and classify the organic residues on a metal surface accurately, we developed a classification model using VNIR HSI technology and machine learning methods. We implemented deep learning methods, such as U-net and CNN-1D, to generate a mask image in the classification model. Owing to the enhanced ROI segmentation and fine-tuned parameters in the CNN layers, both the deep learning methods demonstrated improved classification accuracies in the case of diluted residues. The two mask image algorithms, such as Otsu and PCA, used an optimal thresholding based on a single intensity threshold, which is calculated by the difference between the inter-class variance and between-class variance or loading vector, respectively. These two methods use a single intensity threshold so as to have a fast, simple technique, whereas they tend to find it difficult to separate detailed image parts from background. In turn, U-net adopted encoding and decoding procedures for data reduction and optimal feature selection. Furthermore, data augmentation using the annotated images demonstrated a precise feature segmentation [24]. Typically, organic residues can potentially generate biofilms that cause extracellular polymeric substance production and maturation [33]. Therefore, this study can potentially afford the early detection of biofilms in food processing machines using VNIR HSI and machine learning at an accuracy of *A* = 0.94. However, further research is necessary to obtain diverse evidence to fine-tune the hyperparameters of the deep learning methods, particularly when multiple samples are considered. Additionally, the proposed model must be validated across areas and in more practical locations of the food industry.

## Figures and Tables

**Table 1:** Convolutional neural network and parameters.

Layer	Type	Output Shape	# of Parameter
Conv1d_1	Conv1D	124, 40	240
Average_pooling1d_1	AveragePooling1D	62, 40	0
Max_pooling1d_1	MaxPooling1D	31, 40	0
Conv1d_2	Conv1D	29, 20	2420
Average_pooling1d_2	AveragePooling1D	14, 20	0
Max_pooling1d_2	MaxPooling1D	7, 20	0
Flatten_1	Flatten	140	0
Dense_1	Dense	500	70,500
Dropout_1	Dropout	500	0
Dense_2	Dense	100	50,100
Dense_3	Dense	7	707
	Total parameters: 123,967, Trainable parameters: 123,967		

**Table 2:** Result of the classification of the diluted residues of potato as the accuracy (*A*) and kappa coefficient (*K*) based on chemometric methods.

MA	PP	Hundred	Twenty	Ten	Five	Two	One	BG	A	K
LDA	NoP	1.00	0.87	0.70	0.68	0.60	0.86	1.00	0.83	0.80
	D1	1.00	0.87	0.70	0.68	0.60	0.86	1.00	0.83	0.80
	D2	1.00	0.87	0.70	0.68	0.60	0.86	1.00	0.83	0.80
	MSC	0.98	0.85	0.78	0.39	0.42	0.58	0.82	0.71	0.66
	MA	1.00	0.87	0.70	0.68	0.60	0.86	1.00	0.83	0.80
	NM	1.00	0.86	0.71	0.66	0.62	0.86	1.00	0.83	0.80
PLSDA	NoP	0.99	0.73	0.22	0.63	0.36	0.54	1.00	0.68	0.62
	D1	0.99	0.64	0.01	0.71	0.19	0.07	1.00	0.57	0.49
	D2	1.00	0.56	0.05	0.59	0.37	0.08	1.00	0.58	0.50
	MSC	0.99	0.80	0.67	0.70	0.25	0.65	0.96	0.60	0.52
	MA	0.99	0.73	0.22	0.63	0.36	0.54	1.00	0.68	0.62
	NM	1.00	0.75	0.24	0.67	0.36	0.56	1.00	0.69	0.63
SVM	NoP	1.00	0.89	0.87	0.93	0.71	0.94	0.95	0.90	0.88
	D1	1.00	0.85	0.78	0.79	0.63	0.93	0.95	0.86	0.83
	D2	1.00	0.86	0.71	0.82	0.60	0.92	0.95	0.85	0.82
	MSC	1.00	0.83	0.78	0.78	0.39	0.60	0.94	0.78	0.74
	MA	1.00	0.87	0.71	0.81	0.36	0.90	0.95	0.81	0.78
	NM	1.00	0.87	0.71	0.81	0.36	0.90	0.95	0.81	0.78
DT	NoP	1.00	0.73	0.56	0.68	0.40	0.87	0.95	0.76	0.71
	D1	0.96	0.65	0.37	0.66	0.33	0.88	0.95	0.71	0.65
	D2	0.99	0.70	0.32	0.61	0.31	0.90	0.95	0.70	0.65
	MSC	0.99	0.72	0.69	0.74	0.14	0.46	0.87	0.68	0.62
	MA	1.00	0.73	0.56	0.68	0.40	0.87	0.95	0.76	0.71
	NM	1.00	0.73	0.56	0.68	0.40	0.87	0.95	0.76	0.71
LSSVM	NoP	1.00	0.87	0.81	0.90	0.74	0.95	0.95	0.89	0.87
	D1	1.00	0.86	0.66	0.81	0.67	0.98	0.95	0.85	0.83
	D2	1.00	0.79	0.10	0.62	0.33	0.89	0.95	0.69	0.64
	MSC	0.99	0.80	0.67	0.70	0.25	0.65	0.96	0.74	0.69
	MA	1.00	0.88	0.81	0.88	0.73	0.94	0.95	0.89	0.87
	NM	1.00	0.87	0.78	0.87	0.69	0.94	0.95	0.88	0.86
RF	NoP	0.98	0.93	0.83	0.77	0.67	0.88	1.00	0.88	0.86
	D1	0.98	0.92	0.73	0.76	0.74	0.94	1.00	0.88	0.86
	D2	0.99	0.90	0.58	0.72	0.67	0.95	1.00	0.85	0.82
	MSC	0.98	0.91	0.72	0.59	0.43	0.49	0.99	0.76	0.72
	MA	0.98	0.93	0.83	0.77	0.67	0.88	1.00	0.88	0.86
	NM	0.98	0.88	0.81	0.76	0.67	0.88	1.00	0.87	0.84

MA: multivariate analysis; PP: preprocessing method; No-P: no-preprocessing; D1: 1st derivative; D2: 2nd derivative; MSC: multiplicative scatter correction; MA: moving average; NM: normalization; Hundred: 100% residue; Twenty: 20% diluted residue (1:5 dilution); Ten: 10% diluted residue (1:10 dilution); Five: 5% diluted residue (1:20 dilution); Two: 2% diluted residue (1:50 dilution); One: 1% diluted residue (1:100 dilution); A: Accuracy; K: kappa coefficient.

**Table 3:** Result of the classification of the diluted residues of spinach as the accuracy (*A*) and kappa coefficient (*K*) based on chemometric methods.

MA	PP	Hundred	Twenty	Ten	Five	Two	One	BG	A	K
LDA	NoP	1.00	0.92	0.83	0.77	0.77	0.74	1.00	0.88	0.86
	D1	1.00	0.92	0.83	0.77	0.77	0.74	1.00	0.88	0.86
	D2	1.00	0.92	0.83	0.77	0.77	0.74	1.00	0.88	0.86
	MSC	0.99	0.83	0.90	0.67	0.69	0.43	1.00	0.81	0.78
	MA	1.00	0.92	0.83	0.77	0.77	0.74	1.00	0.88	0.86
	NM	1.00	0.92	0.82	0.78	0.77	0.76	1.00	0.88	0.86
PLSDA	NoP	1.00	0.96	0.87	0.37	0.66	0.74	1.00	0.83	0.80
	D1	1.00	0.93	0.87	0.09	0.46	0.56	1.00	0.75	0.70
	D2	1.00	0.96	0.88	0.18	0.60	0.70	1.00	0.80	0.76
	MSC	1.00	0.86	0.73	0.75	0.56	0.74	0.97	0.81	0.78
	MA	1.00	0.96	0.87	0.37	0.66	0.74	1.00	0.83	0.80
	NM	1.00	0.97	0.87	0.38	0.65	0.74	1.00	0.83	0.80
SVM	NoP	1.00	0.96	0.90	0.88	0.81	0.85	0.98	0.92	0.91
	D1	1.00	0.90	0.76	0.82	0.76	0.78	0.98	0.87	0.85
	D2	1.00	0.92	0.80	0.84	0.79	0.81	0.98	0.89	0.87
	MSC	1.00	0.84	0.78	0.79	0.77	0.72	0.98	0.86	0.83
	MA	1.00	0.76	0.64	0.71	0.76	0.71	0.98	0.81	0.78
	NM	1.00	0.76	0.64	0.71	0.76	0.71	0.98	0.81	0.78
DT	NoP	1.00	0.77	0.63	0.65	0.65	0.68	0.98	0.79	0.75
	D1	1.00	0.85	0.71	0.74	0.60	0.78	0.98	0.83	0.80
	D2	1.00	0.94	0.70	0.84	0.62	0.71	0.98	0.84	0.82
	MSC	1.00	0.89	0.72	0.80	0.72	0.66	0.98	0.84	0.81
	MA	1.00	0.77	0.63	0.65	0.65	0.68	0.98	0.79	0.75
	NM	1.00	0.77	0.63	0.65	0.65	0.68	0.98	0.79	0.75
LSSVM	NoP	1.00	0.89	0.82	0.79	0.70	0.75	0.98	0.86	0.84
	D1	1.00	0.86	0.72	0.64	0.55	0.75	0.98	0.81	0.77
	D2	1.00	0.90	0.73	0.71	0.47	0.70	0.98	0.81	0.77
	MSC	1.00	0.86	0.73	0.75	0.56	0.74	0.97	0.82	0.79
	MA	1.00	0.85	0.79	0.73	0.71	0.73	0.98	0.84	0.81
	NM	1.00	0.90	0.85	0.77	0.75	0.76	0.98	0.87	0.85
RF	NoP	1.00	0.86	0.84	0.78	0.82	0.84	1.00	0.89	0.87
	D1	1.00	0.86	0.81	0.85	0.85	0.86	1.00	0.90	0.88
	D2	1.00	0.89	0.81	0.88	0.79	0.76	1.00	0.89	0.87
	MSC	1.00	0.87	0.84	0.87	0.92	0.85	1.00	0.91	0.90
	MA	1.00	0.86	0.84	0.78	0.82	0.84	1.00	0.89	0.87
	NM	1.00	0.88	0.82	0.79	0.82	0.83	1.00	0.89	0.87

MA: multivariate analysis; PP: preprocessing method; No-P: no-preprocessing; D1: 1st derivative; D2: 2nd derivative; MSC: multiplicative scatter correction; MA: moving average; NM: normalization; Hundred: 100% residue; Twenty: 20% diluted residue (1:5 dilution); Ten: 10% diluted residue (1:10 dilution); Five: 5% diluted residue (1:20 dilution); Two: 2% diluted residue (1:50 dilution); One: 1% diluted residue (1:100 dilution); A: Accuracy; K: kappa coefficient.
